# Breast Milk and Gut Microbiota in African Mothers and Infants from an Area of High HIV Prevalence

**DOI:** 10.1371/journal.pone.0080299

**Published:** 2013-11-26

**Authors:** Raquel González, Inácio Mandomando, Victoria Fumadó, Charfudin Sacoor, Eusébio Macete, Pedro L. Alonso, Clara Menendez

**Affiliations:** 1 Barcelona Centre for International Heath Research (CRESIB, Hospital Clínic -Universitat de Barcelona), Barcelona, Spain; 2 Manhiça Health Research Centre (CISM), Maputo, Mozambique; 3 Instituto Nacional de Saúde, Ministério de Saúde, Maputo, Mozambique; 4 Sant Joan de Déu Paediatrics Hospital, Barcelona, Spain; University of South Florida College of Medicine, United States of America

## Abstract

**Background:**

Human milk and infant gut microbiota are essential for the immune system maturation and protection against infections. There is scarce information on the microbiological composition of breast milk in general, and none from developing countries. The objective of the study was to characterize the breast milk and gut microbiota from mothers and infants from southern Mozambique, where infections and breastfeeding are prevalent.

**Methods:**

A community-based study was undertaken among 121 pairs of women and infants. Breast milk and infant's faeces were analyzed by bacterial culture and molecular methods. Breast milk samples were screened for HIV RNA by RT-PCR.

**Results:**

The most frequent bacterial groups isolated by culture media in breast milk were *Staphylococci* (96.4%), *Streptococci* (92.7%) and *Lactobacilli* (56.4%). HIV RNA was detected in 24% of the samples. *Staphylococcus hominis*, *S. aureus*, and *S.parasanguis* were more frequently isolated in infants ≤14 days of life. Women on exclusive breastfeeding presented higher proportion of *S. parasanguis* in breast milk than those on mixed infant feeding (36.4% *versus* 11.1%, p = 0.035). Bacterial diversity (mean number of bacterial species isolated by sample: 10.4 *versus* 8.5; p = 0.004) and the frequency of *Lactobacillus spp* (75.9% versus 36%, p = 0.003) were higher in the specimens with HIV RNA than in those without it. The main bacterial groups found in infant's faeces were *Bifidobacterium*, *Streptococci* and *Enterococci*.

**Conclusions:**

Women with HIV RNA in breast milk had a different pattern of microbiological composition, suggesting specific immunopathological phenomena in HIV-infected women. Both breast milk and faecal microbiota composition varied with lactation period, which might be related to changes in the type of feeding over time and/or in the milk's biochemical characteristics. These findings provide insights into interactions between commensal bacteria and HIV infection in human milk and the role of these bacteria in mucosal protection against infections in breastfed infants.

## Introduction

Breastfeeding is the optimal form of infant's feeding since human milk contains all the nutrients needed in the first 6 months of life [1]. Apart from this nutritive role, human milk also influences the development of the immune system through intestinal microbe colonisation [2], [3]. Moreover, some microbiological strains isolated in breast milk samples have probiotic properties [4]. It has been shown that the natural microbiota of the human mammary gland is composed by staphylococci, streptococci, lactic acid bacteria (LAB), propionibacteria and closely related Gram-positive bacteria, and bifidobacteria [5], some of which such as *Lactobacillus gasseri* and *Enterococcus faecium* can potentially prevent infections in breastfeeding infants [6], [7]. Similarly, bifidobacteria and LAB are used as probiotics, and the efficacy of the latter to treat lactational mastitis has recently been shown [8], [9]. *In vitro* studies have also shown that commensal LAB from human breast milk inhibit HIV-1 virus, suggesting a possible role of these bacteria in mucosal protection against HIV in the exposed breastfed infant [5], [10]. Furthermore, in HIV-infected children probiotics may restore the microbiota, increase naïve CD4 T-cell counts, and protect against inflammation and chronic immune activation of the gastrointestinal immune system [11], [12].

Gastrointestinal microbiota also constitutes a key determinant of the host's health, and its diversity, complexity and relationship with human milk microbiota have been described [13], [14]. Moreover, the mode of delivery and the type of feeding method influence the infant's gut microbiota [15]. Despite all the information on the potential influence of the breast milk and gut microbiota on the host health, there is very limited information on the composition of commensal and potentially probiotic bacteria in the human milk of healthy women. This may be particularly important in developing countries, where infections are frequent and breastfeeding is common [16]–[18].

The objective of the study was to describe the microbiological composition of breast milk in women and their infant's gut in a population exposed to high prevalence of infections.

## Methods

### Ethics Statement

The study protocol and the informed consent form were reviewed and approved by the National Committee on Health Bioethics of Mozambique and the Ethics Committee of the Hospital Clínic in Barcelona, Spain. Written informed consent was obtained from all study participants. Informed consent from legal guardians of minors participating in the study was not specifically requested. Study participants were all breastfeeding mothers who were considered autonomous and independent to decide on their participation on the study, according to the aforementioned Ethics Committees that reviewed and approved the informed consent procedures.

### Study site and population

The study was conducted in Manhiça, Maputo province, in southern Mozambique between April and May of 2006. The Centro de Investigação em Saúde de Manhiça (CISM) runs a Demographic Surveillance System (DSS) in the area that covers Manhiça town and the surrounding villages with a total population of around 90.000 inhabitants. The study area and population characteristics have been described in detail elsewhere [19], [20]. At the time the study was conducted, the estimated HIV prevalence among pregnant women was 23% [21]. Prevalence of exclusive breastfeeding in children less than 6 months of age was 5.3%, almost 30% of infants received liquids other than breast milk before 1 week of age, and the mean duration of breastfeeding was 19 months (R González, unpublished data from 2004).

### Study design

This is a cross-sectional, community-based descriptive study of the microbiological composition of breast milk from apparently healthy women and the faeces of their breastfeeding infants. Women who delivered the previous year were randomly selected from a list generated by the CISM DSS census. Four age groups of infants (corresponding to 4 lactation periods) were defined in order to have a wider representation of the microbiota in women's breast milk and infant's faeces: 1) ≤14 days; 2) between 15 and 90 days; 3) between 91 and 180 days, and 4) from 181 to 360 days of life. The target sample size was 30 pairs of mothers and infants per age group.

### Recruitment and sample collection

Study candidates were visited at home by a field worker who explained the study objectives and procedures. Inclusion criteria for women were being resident in the study area, having given birth in the previous 12 months a living child currently being breastfed, and signing the study informed consent (following counselling). Demographic, nutritional data, and information of breastfeeding practices, history of known diseases, and current treatments from the mother and infant were collected onto standardised questionnaires. The baby's birth weight was collected from the infant's health card. The maternal nutritional status was assessed by measuring the mid upper arm circumference (MUAC). Study infants were weighed using a digital scale. Five mL breast milk samples were collected into sterile tubes. Study women were asked to discard the first 2 drops of milk and to fill in the tubes directly by manual expression. A plastic container was delivered to the mothers for collection of the infant's faeces on the day after the household visit. Breast milk and faecal samples were transported at 4°C in a cool box until delivery to the laboratory where they were stored at −80°C until analyses were performed.

### Laboratory methods

Whole breast milk samples were screened for HIV RNA using the QIA UltraSens Viral RNA Isolation kit (Qiagen) and by RT- PCR (Applied Bisosystem). Positive samples were confirmed using a nested HIV-1 LTR (Long Terminal Repeat) PCR. A complete description of the bacterial diversity was performed by culture-based methods in a randomly selected sub-sample (n = 55) of milk specimens from mothers of the four pre-defined lactation periods. Proper peptone water dilutions of the milk samples were plated in duplicate onto Brain Heart Infusion (BHI, Oxoid, Basingstoke, UK; a general-purpose medium suitable for the cultivation of non-fastidious bacteria, yeasts and moulds), MacConkey agar (MCK, BioMerieux; a selective medium for the isolation of enterobacteria) and Columbia Nadilixic Acid Agar (CNA, BioMerieux; a highly nutritious, general-purpose medium for the isolation and cultivation of fastidious microorganisms) agar plates which were aerobically incubated at 37°C for up to 48 h. In parallel, the same samples were also cultured on the Man, Rogosa, and Sharpe (MRS, Oxoid; a medium for the isolation of lactic acid bacteria and bifidobacteria) agar plates, supplemented with cysteine (0.5% w/v) (MRS-Cys). The selected isolates were observed by optical microscopy to determine their morphology and Gram staining. Additionally, they were tested for catalase, oxidase and coagulase activities and for growth on plates of two selective media: Baird-Parker (BP, BioMerieux; a selective medium for the isolation of staphylococci) and Kanamycin Aesculin-Azide Agar (KAA, Oxoid; a selective medium for the isolation of enterococci). Final identification of the isolates was done by PCR sequencing of a 470 pb fragment of the 16S RNA gene.

Following culture-based bacterial identification, milk and faecal samples (N = 120) underwent bacterial DNA isolation for subsequent characterization by quantitative real-time PCR (qRTi-PCR). Briefly, a fraction of the milk sample (1 mL) was initially centrifuged at 7.150×*g* for 20 min. Then, total DNA from the milk pellets and from the infant faeces (500 mg) was isolated using the QIAampDNA Stool Mini Kit (QIAgen, Hilden, Germany). DNA was eluted in 20 µl of buffer AE (provided in the kit), and the purified DNA extracts were stored at −20°C. PCR amplification and detection were performed on optical-grade 96-well plates using an iQ5 Cycler Multicolor real-time PCR detection system (Bio-Rad Laboratories, Hercules, CA) and universal primers.

### Data management, definitions and statistical analysis

Study questionnaires were double entered using the Microsoft® Visual FoxPro 5.0 software (Microsoft Corporation, Redmond, WA, USA). Statistical analysis was performed using STATA statistical software version 11 (STATA Corp., College Station, Texas, USA). Samples found PCR positive but not quantifiable (numeric result under the PCR detection limit) were codified as equal to 1 log genome equivalents/mL for the analysis. Infant's nutritional status was assessed using weight for age Z-score. Malnutrition was defined as a weight for age (WAZ) Z-score lower than -1. In addition, mild malnutrition was defined as a WAZ Z-score between -2 and -1; moderate malnutrition between -3 and -2; and severe malnutrition <-3. The effect of breastmilk's HIV RNA, infant's type of breastfeeding (exclusive or mixed), lactation period, nutritional status, and mother's gravidity on milk and faecal bacterial counts was analysed using uncorrected χ^2^ test, Fisher exact test, t-Student or ANOVA test according to variable characteristics.

## Results

### Study participants

A total of 144 households were visited and 140 women were finally seen and invited to participate in the study. Of them, 121 accepted to participate in the study (acceptance rate 86.4%). A total of 121 breast milk and 120 faecal samples were collected. The demographic characteristics of study participants are presented in **Table 1**. Four women reported being on medical treatment (amoxicilline, paracetamol, cotrimoxazole and antihypertensive drugs respectively) at the time of the household visit. The proportion of infants receiving exclusive breastfeeding at the time of the interview differed among the four age groups: 70% in those aged ≤14 days, 33.3% in those aged 15 to 90 days, 10% in those aged 91 to 180 days and 3.2% in infants aged 6 to 12 months. The proportion of exclusive breastfeeding in infants aged less than 6 months was 37.8%.

**Table 1:** Characteristics of study participants (n = 121).

Women's age (years)	Mean (SD)	25.2 (6.5)
	Median (range)	24.8 (15 – 47)
MUAC* (cm)	>22	119 (98.4)
	≤22	2 (1.6)
Type of delivery	Vaginal	116 (95.9)
	Caesarian	5 (4.1)
Gravidity	1	35 (28.9)
	2 to 3 pregnancies	46 (38.0)
	4 or more	40 (33.0)
Infant's age groups (n)	≤2 weeks	30
	>2 weeks-≤3 months	30
	>3 months-≤6 months	30
	>6 months-≤12 months	31
Infants' sex ♂		68 (56.2)
Exclusive breastfeeding		35 (28.9)
Birthweight (g) Mean (SD)		3007.19 (467.22)
Infant's Malnutrition	Mild	27 (22.1)
	Moderate	9 (7.4)
	Severe	1 (0.8)

Values are number n (%) unless indicated otherwise

* Mid-Upper Arm Circumference

### Bacterial analysis of breast milk samples

The 55 breast milk samples analysed yielded a positive culture for BHI and CNA culture media. The mean value of total bacterial counts in breast milk samples ranged from 1.20 to 5.50 log10 CFU/mL and differed among culture media (3.54 in BHI medium, 3.86 in CNA and 2.88 in MRS-Cys). No colony was found in 5 samples when plated in MRS-Cys (for isolation of lactobacilli). The bacterial diversity was wide and included 44 genera and 124 species. The mean number of species isolated by sample was 9.5 and ranged from 4 to 14. The three most frequent bacterial genera isolated by culture were *Staphylococci* (96.4% of 55 samples), *Streptococci* (92.7%) and *Lactobacilli* (56.4%) (**Table 2**). Bifidobacteria were detected in 10.9% (6/55) of the samples. The results of the breast milk analyses by qRTi-PCR are shown in Table 2. The most frequent groups identified by PCR were *Streptococci* (94.8%), *Staphylococcus epidermidis* (85.4%), *Enterococci* (83.9%) and *Bifidobacterium* (81.8%). *Staphylococcus hominis*, *Staphylococcus aureus*, and *Streptococcus parasanguis* were more frequently isolated in infants aged less than 15 days of age than in the other lactation periods (**Table 3**). Breast milk samples from women who reported exclusive breastfeeding presented higher proportion (36.4%) of *S. parasanguis* in breast milk than those from women who reported mixed infant feeding (11.1%, p = 0.035). This difference with respect to the type of infant breastfeeding was not found for the rest of the bacterial isolates in milk samples. Mother's gravidity and infant's nutritional status were not associated with milk bacterial counts (p>0.05).

**Table 2:** Frequency of main bacterial genus, groups and species isolated in breast milk samples by culture and qRTi-PCR.

Bacterial Group	Culture			qRTi-PCR		
	n/55	%	Bacterial Count^1^ m (SD)	n/N	%	Bacterial DNA^2^ m (SD)
*Staphylococcus*	53	96.4				
* S. epidermidis*	48	87.3	3.38 (2.93)	82/96	85.4	3.46 (1.55)
* S. hominis*	44	80.0	2.76 (2.42)	ND	ND	ND
* S. aureus*	20	36.4	1.02 (1.72)	40/95	42.11	1.40 (1.67)
* Streptococcus*	51	92.7	ND	91/96	94.8	3.10 (1.00)
* S. salivarius*	33	60	1.67 (1.99)	ND	ND	ND
* S. mitis*	33	60	1.11 (1.24)	ND	ND	ND
* S. parasanguis*	11	20	0.51 (1.17)	ND	ND	ND
* Strept. sp*	43	78.2	2.41 (2.07)	ND	ND	ND
*Lactobacillus*	31	56.4	ND	78/109	71.6	2.69 (1.65)
* L. gastricus*	19	34.6	0.75 (1.44)	ND	ND	ND
* L. fermentum*	8	14.6	0.25 (0.67)	ND	ND	ND
* L. gasseri*	9	16.4	0.25 (0.64)	ND	ND	ND
* Lactob. sp*	14	25.5	0.51 (1.03)	ND	ND	ND
* Pediococcuspentosaceus*	7	12.7	0.31 (1.39)	ND	ND	ND
*Bifidobacterium*	6	10.9	0.18 (0.64)	99/121	81.8	4.82 (0.65)
*Kocuri arhizophila*	17	30.9	0.45 (0.81)	ND	ND	ND
*Rothia mucilaginosa*	13	23.6	0.38 (0.78)	ND	ND	ND
*Gemella haemolysans*	12	21.8	0.27 (0.59)	ND	ND	ND
*Other species*	51	92.7	4.6 (4.04)	ND	ND	ND
*Enterococcus*	ND	ND	ND	89/106	83.9	3.11 (0.70)
*Bacteroides*	ND	ND	ND	13/103	12.6	0.61 (1.11)
*Clostridium leptum*	ND	ND	ND	21/96	21.9	0.78 (1.43)
*Clostridium coccoides*	ND	ND	ND	69/95	72.6	2.04 (1.31)

1 Data are expressed as log_10_ colony-forming units/mL;^2^Bacterial DNA expressed as log genome equivalents/mL; ND: Not determined.

**Table 3:** Isolation of bacterial groups and RNA HIV in breast milk by lactation period.

Bacterial Group	Lactation period								
	(≤14 days) N = 15		(15–90 days) N = 10		(91–180 days) N = 16		(≥181 days) N = 14		P*
	n	%	n	%	n	%	n	%	
*Staphylococcus*	15	100	10	100	14	87.5	14	100	0.243
* S. epidermidis*	14	93.3	10	100	12	75.0	12	85.7	0.322
* S. hominis*	15	100	10	100	9	56.3	10	71.4	0.003
* S. aureus*	11	73.3	3	30	2	12.5	4	28.6	0.004
*Streptococcus*	14	93.3	9	90	16	100	12	85.7	0.458
* S. salivarius*	8	53.3	5	50	12	75	8	57.1	0.554
* S. mitis*	7	46.7	7	70	10	62.5	9	64.3	0.677
* S. parasanguis*	9	60	2	20	0	0	0	0	0.001
* Strept. sp*	9	60	8	80	15	93.8	11	78.6	0.161
*Lactobacillus*	5	33.3	6	60	12	75	8	57.1	0.146
HIV RNA	6/30	20	4/30	13.3	11/30	36.7	8/31	25.8	0.204

* Fisher exact test p<0.05.

n = number of samples where the bacterial group or HIV RNA was detected.

### HIV RNA and microbiota in breast milk

HIV RNA was detected in 29 out of 121 (24.2%) milk samples. HIV-1 viral load ranged from 1,215 to 125,565 copies/mL. Though not statistically significant, the proportion of reported exclusive breastfeeding tended to be lower (6/29 [20.7%]) in women where HIV RNA was detected in milk than in those where the virus was not detected (28/91 [30.77%], p = 0.16). There was not an obvious trend between HIV detection in breast milk samples and the lactation period (**Table 3**).

**Figure 1:**
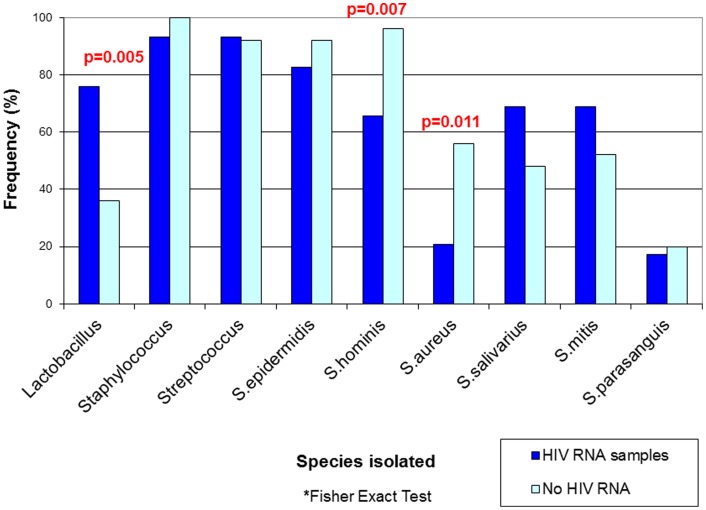
Frequency of main bacterial species isolated in breast milk samples by culture medium and presence of HIV RNA. *Fisher Exact Test.

The mean value of total bacterial counts in the three culture media were not different in samples where HIV RNA was detected compared to those where it was not detected. However, the mean number of bacterial species isolated by sample was significantly higher in those specimens with HIV RNA (10.4 *versus* 8.5 species/sample, p = 0.004). The frequency of *Lactobacillus* was also higher (75.9% versus 36.0%, p = 0.005) in milk samples where HIV RNA was detected compared to those with no HIV RNA detection. In contrast, the frequencies of *S. hominis* and *S. aureus* were statistically significantly reduced in samples with detected HIV RNA (65.5% versus 96.0% [p = 0.007] and 20.7% versus 56.0% [p = 0.011], respectively, **Figure 1** and **Table S1**).

### Composition of infant's faecal microbiota

**Figure 2:**
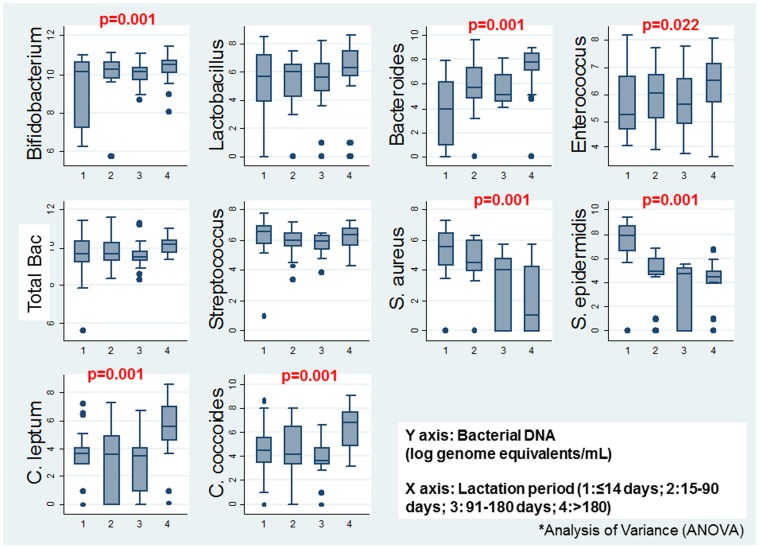
Mean bacterial DNA detected in faecal samples by infant lactation period. *Analysis of Variance (ANOVA).

**Figure 3:**
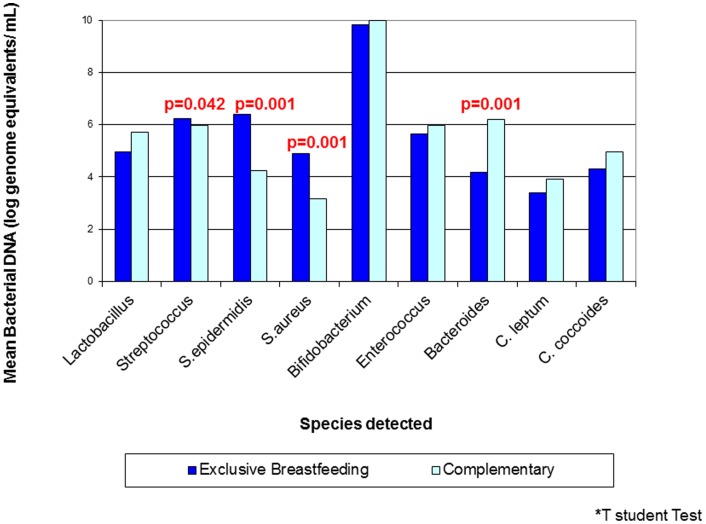
Mean bacterial DNA detected in faecal samples by type of infant breastfeeding. *T Student test.

**Table 4** shows the frequencies and mean bacterial DNA of bacterial species detected by PCR in infant's samples. *Streptococci, Enterococci,* and *Bifidobacteria* groups were detected in all samples analysed, while *Staphyloccoccus* was the bacterial group least detected (76.7%). Bacterial DNA concentrations differed by lactation period. *Bifidobacterium, Bacteroides, Enterococcus, Clostridium leptum* and *Clostridium coccoides* counts increased with infant's age whereas *S. aureus* and *S. epidermidis* counts were significantly reduced in older infants (**Figure 2** and **Table S2**). Mean bacterial DNA concentration of *Streptococcus spp., S. epidermidis* and *S. aureus* was statistically significantly higher in faecal samples from infant's whose mothers reported exclusive breastfeeding than in those who did not (p<0.05, **Figure 3** and **Table S3**). These differences by type of infant breastfeeding were not found for the rest of the bacterial groups, except for *Bacteroides* group, that presented a higher concentration in women reporting complementary feeding (**Figure 3** and **Table S3**). No differences were found on faecal bacterial counts by presence of HIV RNA in milk andmother's gravidity (data not shown). On the other hand, bacterial DNA concentrations of *Streptcoccus, Clostridium leptum* and *Clostridium coccoides* were increased in malnourished infants **(Table S4**). Finally, there was a correlation between maternal milk and infant's faecal microbiota for *S. epidermidis* detected by PCR (p = 0.002).

**Table 4:** Frequency of main bacterial genus, groups and species detected in faecal samples by qRTi-PCR.

Bacterial Group		qRTi-PCR (N = 120)		
		n	%	Bacterial DNA m (SD)
*Streptococcus*		120	100	6.05 (0.90)
*Bifidobacterium*		120	100	9.94 (1.11)
*Enterococcus*		120	100	5.87 (1.09)
*Clostridium coccoides*		117	97.5	4.77 (1.94)
*Lactobacillus*		114	95	5.51 (1.96)
*Bacteroides*		111	92.5	5.63 (2.48)
*Staphylococcus*	*S.epidermidis*	103	85.3	4.88 (2.51)
	*S.aureus*	92	76.7	3.69 (2.35)
*Clostridium leptum*		101	84.2	3.77 (2.31)

Bacterial DNA expressed as log genome equivalents/Ml.

n =  number of samples where the bacterial group was isolated.

## Discussion

This is to our knowledge the first detailed description of breast milk microbiota in women from developing countries, and the first study on the faecal microbiota of apparently healthy African infants from the community in contrast to previous hospital-based studies [22], [23]. Moreover, it is the first characterization of the milk bacterial composition in HIV-infected women. The main findings show that women with HIV RNA in breast milk have a different pattern of microbiological composition with increased bacterial diversity and lactobacillus in their milk, suggesting specific immunopathological phenomena in HIV-infected women.

The mechanism by which HIV RNA in breast milk is associated with increased diversity of bacterial species and enhancement of LAB overgrowth is difficult to elucidate. It could be speculated that the associated HIV immunosuppression may favour specific bacterial colonization. On the other hand, it might be hypothesized that LAB species are increased in breast milk from HIV-infected women due to changes in milk components such as oligosaccharides, which are suggested to act as prebiotics in the selection of gut's microbiota [24]. Infact, it has been showed that human milk oligosaccharides have a function that drives the intestinal microbiota toward bifidogenic population[25]. The increased presence of LAB in breast milk of HIV-infected women is especially relevant since these bacteria have the potential for protection against infections [26]. Moreover, a recent in vitro study has shown that breast milk commensal LAB inhibit HIV-1 could prevent access of the virus to cells lining the infant's gastrointestinal tract [10]. In contrast, *S.aureus* and *S.hominis* were significantly reduced in samples from women with breast milk HIV RNA detected. This might be explained by the reported growth inhibition of *S.aureus* and other microorganisms by LAB and Bifidobacteria [26]. Noticeably, the bacterial composition of infant's faeces was not modified by the presence of HIV RNA in breast milk samples, suggesting that the potential influence of the HIV virus on commensal bacteria might be explained by immune or biochemical factors in the milk and that it would not translate into effects on the infant's gut microbiota. The observed differences in the microbiological pattern between samples with and without HIV RNA detected in the breastmilk might also be explained by differences in nutritional habits or socio-economic characteristics of the two group of women.

Exclusive breastfeeding was less common among women in whom HIV RNA was detected in breast milk. This is of concern since the introduction of food proteins or enteric pathogens during formula or mixed replacement feeding may increase the likelihood of HIV transmission by stimulating intestinal inflammatory responses in the neonatal gut, leading to increased permeability and to facilitation of virus penetration [27]. This might also be explained by socio-economic differences between HIV infected and non-infected women. Interestingly, in this community-based study the proportion of reported exclusive breastfeeding in infants aged less than 6 months was 37.5% which is similar to rates reported in Tanzania and Uganda [28], [29]. However, these results are in contrast to those of a community-based study conducted previously in the same area where such proportion was 5.3% (unpublished data from 2004). These findings suggest an increase of the rates of exclusive breastfeeding among mothers from the study area in last years that could be explained by a possible effect of the recent sensitization campaigns promoting exclusive breastfeeding at the antenatal clinic level. Otherwise, such discrepancies between the two studies could be due to a maternal recall bias with age or differences in procedures between studies [30].

In breast milk, *Staphylococcus epidermidis, Streptococcus spp.* and LAB species were the main bacterial groups isolated by culture, while by molecular analysis *Streptococcus* bacteria showed the highest frequencies followed by *Staphylococci, Enterococci, Bifidobacterium* and LAB groups. These findings are similar to those from studies conducted among European women in whom *Staphylococci, Enterococci, Lactococci* and LAB were the most frequent bacterial species isolated in breast milk [7], [14], [26]. These results suggest that environmental factors may not greatly influence the bacterial composition of human milk. Earlier studies conducted in West Africa also found similarities in breast milk fat concentration between Gambian and British mothers despite significant differences in the nutritional components of their diet [31].

Interestingly, some frequencies of bacterial counts differed among lactation period. In particular, *S. aureus, S.hominis* and *S.parasanguis* were more frequent in infants aged ≤14 days old (first lactation period) than in older infants, highlighting the specific characteristics of the human milk composition in the first weeks of breastfeeding. Indeed, breast milk in the first two weeks after delivery is known to have unique properties. Colostrum, which is secreted in the first 2–3 days after delivery, contains a higher concentration of proteins, minerals and fat-soluble vitamins (A, E and K) than later milk [1]. In fact, breast milk is only considered “mature” after two weeks of delivery, and it could be hypothesized that the characteristics of this “early” milk rich in oligosaccharides may favour commensal colonisation by some bacteria species. A study conducted among 18 mothers has also found that the human milk microbiome changes over lactation [32]. The type of infant breastfeeding might also explain the bacterial composition of human milk in the first weeks after delivery, since the proportion of exclusive breastfeeding in infant's aged ≤14 days was the highest (70%) compared to other lactation periods.

*Streptococci, Enterococci* and *Bifidobacterium* were the genera present in all faecal samples analysed by molecular analysis. A study among European breastfed neonates also reported the predominance of bifidobacterial species in the infant's gut [13]. Faecal microbiota from the Mozambican infants in this study showed an abundance of *Bifidobacterium* DNA, which is in accordance with results from studies conducted among Malawian 6-month-old infants [22]. In the current study, the infant's lactation period seemed to influence their bacterial faecal composition. The faecal DNA concentration of *Bifidobacterium*, *Bacteroides* and *Enterococcus* where higher in older infants than in those aged ≤14 days. It cannot be ruled out that the type of infant's breastfeeding could explain this trend, since the proportion of exclusive breastfeeding significantly decreased with the infant's age. In a study conducted in European infants it was shown that the faecal microbiota after introduction of complementary foods is different to that before weaning [33]. In the present study, faecal DNA concentration for *Streptococcus spp., S.epidermidis* and *S.aureus* groups were significantly higher in exclusively breastfed infants than in those on mixed feeding. These results are in agreement with previous reports where the type of breastfeeding was the main factor determining intestinal colonisation of neonates [26], [34].

Previous studies have shown that the bacterial composition of the gut microbiota of breastfed infants is closely related to that found in the breast milk of their respective mothers [14], [35]. In this study we only found a positive correlation between maternal's milk and infant's faeces for *S.epidermidis* group analyzed by the aforementioned PCR. Interestingly, presence of *S. epidermidis* seems to be a differential trait of the faecal microbiota of breast-fed infants [36]. In contrast, no correlation was found for *Lactobacillus* and *Bifidobacteria* groups. Differences in the populations studied, specifically high exposure of both the mother and the baby to multiple micro-organisms in this population might explain the lack of correlation for most bacterial groups between milk and faecal specimens.

Although not focussed on the bacterial composition, earlier studies showed that breast milk composition is influenced by the parity of the mother with increased fat concentration in breast milk from primiparous mothers [31]. In this study, parity was not associated neither with differences in the breast milk microbiota nor with changes in the infant faecal one.

In addition, an association was found between the gut microbiota and the infant's nutritional status for *Streptococcus, Clostridium leptum* and *Clostridium coccoides* bacterial groups, which were significantly increased in infants with malnutrition. This finding supports that malnutrition may be a possible determinant of faecal microbiota and it could be hypothesized that gut microbiota and malnutrition have a common cause or similar determinants [37], [38].

This study has some potential limitations, such as that the set of primers used in the molecular analysis may not have identified other bacterial groups present in the samples. On the other hand, information on the immunological status of the HIV-infected women, which might help explaining the findings in bacterial milk composition in these women, was not available. In addition, it cannot be excluded that women with undetected HIV RNA in their breast milk were actually infected since the virus may be present as proviral DNA in latent T cells or as intracellular RNA in breast milk, or be undetected in individuals receiving antiretroviral treatment [39], [40]. However, we think that the frequency of HIV RNA detected in milk reflects the true prevalence of HIV in this study. First, at the time of the study, the number of women receiving antiretroviral treatment was very low in this population, and more importantly the prevalence of women with detected HIV RNA in breast milk among study participants was comparable to the prevalence of HIV seropositivity in pregnant women attending the local antenatal clinic [21].

In summary, this study provides a detailed description of the bacterial composition of breast milk and infant's faeces from a population in sub-Saharan Africa with one of the highest world's rates of HIV infection and vertical transmission of HIV. Future research is much needed to elucidate the interactions between commensal bacteria and HIV in breast milk and the role for these bacteria in mucosal protection against the infection in breastfed infants in order to help preventing HIV vertical transmission.

## Supporting Information

Table S1 **Isolation of bacterial groups by presence of RNA HIV in breast milk.** *Fisher exact test p<0.05; n = number of samples where the bacterial group was detected.
(DOCX)

Table S2 **Mean bacterial DNA detected in faecal samples by infant lactation period (n = 120).** *Analysis of Variance (ANOVA).
(DOCX)

Table S3 **Mean bacterial DNA detected (Log genome equivalents/mL) in faecal samples by type of infant breastfeeding (n = 120).** *T Student test p<0.05.
(DOCX)

Table S4 **Mean bacterial DNA detected (Log genome equivalents/mL) in faecal samples by infant nutritional status.** *T Student test p<0.05.
(DOCX)
