# Correction: Breast Milk and Gut Microbiota in African Mothers and Infants from an Area of High HIV Prevalence

**DOI:** 10.1371/journal.pone.0092930

**Published:** 2014-03-13

**Authors:** The PLOS ONE Staff

The laboratory analyses reported in this article were conducted by researchers based at the Department of Nutrition, Food Science and Food Technology, Complutense University of Madrid and the Institute of Clinical and Molecular Virology, University of Erlangen-Nuremberg. These researchers should be included in the author list in the article. The authors apologize for this omission.

The correct author list is:

Raquel González^1,2^*, Antonio Maldonado^3^*, Virginia Martín^3^*, Inácio Mandomando^2,4^, Victoria Fumadó^1,5^, Karin J. Metzner^6,7^, Charfudin Sacoor^2^, Leónides Fernández^3^, Eusébio Macete^2^, Pedro L. Alonso^1,2^, Juan M. Rodríguez^3^®, Clara Menéndez^1,2^®

* These authors equally contributed to this work; ® Corresponding authors.

Affiliations:

1. Barcelona Centre for International Heath Research (CRESIB, Hospital Clínic -Universitat de Barcelona), Barcelona, Spain

2. Manhiça Health Research Centre (CISM), Maputo, Mozambique

3. Department of Nutrition, Food Science and Food Technology, Complutense University of Madrid, Spain

4. Instituto Nacional de Saúde, Ministério de Saúde, Maputo, Mozambique

5. Sant Joan de Déu Paediatrics Hospital, Barcelona, Spain

6. Institute of Clinical and Molecular Virology, University of Erlangen-Nuremberg, Erlangen, Germany

7. Division of Infectious Diseases and Hospital Epidemiology, University Hospital Zürich, University of Zürich, Zürich, Switzerland

The authors from Complutense University of Madrid and Dr Metzner declare that no competing interests exist. The summary of contributions to the study should be revised as below:

Conceived and designed the experiments: RG AM KJM VF PLA JMR CM.

Performed the experiments: RG AM VM IM CS VF LF.

Analyzed the data: RG KJM

Contributed reagents/materials/analysis tools: RG AM VM IM KJM CS EM.

Wrote the paper: RG JMR CM.

Reviewed and approved the last version manuscript: RG AM VM IM VF KJM CS LF EM PLA JMR CM.

The correct citation is: González R, Maldonado A, Martin V, Mandomando I, Fumadó V, et al. (2013) Breast Milk and Gut Microbiota in African Mothers and Infants from an Area of High HIV Prevalence. PLoS ONE 8(11): e80299. doi:10.1371/journal.pone.0080299

The funding statement should also be corrected to acknowledge the grant that supported the authors from Complutense University of Madrid: Laboratorial analyses were funded by a grant from the Spanish Ministry of Economy and Competitiveness (grant AGL2005-01138).
