# Supplementary material for: Breast Milk and Gut Microbiota in African Mothers and Infants from an Area of High HIV Prevalence
Source: PLoS One. 2013 Nov 26;8(11):e80299. doi: 10.1371/journal.pone.0080299 (PMC3841168; doi:10.1371/journal.pone.0080299)
Supplement: Table S1 — Isolation of bacterial groups by presence of RNA HIV in breast milk. *Fisher exact test p<0.05; n = number of samples where the bacterial group was detected. (DOCX) [file pone.0080299.s001.docx]

**Table S1.** Isolation of bacterial groups by presence of RNA HIV in breast milk

| **Bacterial group** | | **RNA HIV** | | **No HIV RNA** | | **P*** |
| --- | --- | --- | --- | --- | --- | --- |
|  |  | **n/29** | **%** | **n/25** | **%** |  |
| *Lactobacillus* | | 22 | 75.9 | 9 | 36.0 | **0.005** |
| *Staphylococcus* |  | 27 | 93.1 | 25 | 100 | 0.493 |
|  | *S. epidermidis* | 24 | 82.8 | 23 | 92.0 | 0.431 |
|  | *S. hominis* | 19 | 65.5 | 24 | 96.0 | **0.007** |
|  | *S. aureus* | 6 | 20.7 | 14 | 56.0 | **0.011** |
| *Streptococcus* |  | 27 | 93.1 | 23 | 92 | 1.000 |
|  | *S. salivarius* | 20 | 69.0 | 12 | 48.0 | 0.167 |
|  | *S. mitis* | 20 | 69.0 | 13 | 52.0 | 0.266 |
|  | *S. parasanguis* | 5 | 17.24 | 5 | 20.0 | 1.000 |

*****Fisher exact test p<0.05

n=number of samples where the bacterial group was detected
