# Supplementary material for: Breast Milk and Gut Microbiota in African Mothers and Infants from an Area of High HIV Prevalence
Source: PLoS One. 2013 Nov 26;8(11):e80299. doi: 10.1371/journal.pone.0080299 (PMC3841168; doi:10.1371/journal.pone.0080299)
Supplement: Table S2 — Mean bacterial DNA detected in faecal samples by infant lactation period (n = 120). *Analysis of Variance (ANOVA). (DOCX) [file pone.0080299.s002.docx]

**Table S2.** Mean bacterial DNA detected in faecal samples by infant lactation period (n=120)

| **Bacterial Group** | | **Lactation period** | | | | | | | |  |
| --- | --- | --- | --- | --- | --- | --- | --- | --- | --- | --- |
|  |  | **(≤ 14 days)** | | **(15- 90 days)** | | **(91-180 days)** | | **(≥181 days)** | | **P*** |
|  |  | **Mean** | **SD** | **Mean** | **SD** | **Mean** | **SD** | **Mean** | **SD** |  |
| *Bifidobacterium* |  | 9.260 | 1.678 | 10.132 | 0.926 | 10.066 | 0.553 | 10.334 | 0.650 | **0.001** |
| *Lactobacillus* |  | 5.270 | 2.281 | 5.237 | 1.876 | 5.356 | 1.747 | 6.197 | 1.842 | 0.180 |
| *Bacteroides* |  | 3.418 | 2.840 | 6.032 | 1.950 | 5.736 | 1.318 | 7.323 | 1.854 | **0.001** |
| *Enterococcus* |  | 5.563 | 1.129 | 5.903 | 1.048 | 5.662 | 1.024 | 6.359 | 1.060 | **0.022** |
| *Streptococcus* |  | 6.292 | 1.223 | 5.932 | 0.867 | 5.766 | 0.590 | 6.225 | 0.734 | 0.076 |
| *Staphylococcus* | *S. epidermidis* | 7.457 | 1.805 | 4.618 | 2.056 | 3.597 | 2.226 | 3.859 | 1.964 | **0.001** |
|  | *S. aureus* | 5.35 | 1.504 | 4.212 | 2.109 | 3.005 | 2.222 | 2.152 | 2.210 | **0.001** |
| *Clostridium leptum* |  | 3.180 | 1.929 | 3.309 | 2.350 | 3.053 | 1.959 | 5.545 | 2.123 | **0.001** |
| *Clostridium coccoides* |  | 4.379 | 1.927 | 4.399 | 1.824 | 3.845 | 1.460 | 6.459 | 1.503 | **0.001** |
| *Total bacteria* |  | 9.646 | 1.059 | 9.807 | 0.691 | 9.608 | 0.626 | 10.079 | 0.442 | 0.061 |

*****Analysis of Variance (ANOVA)
