# Supplementary material for: Breast Milk and Gut Microbiota in African Mothers and Infants from an Area of High HIV Prevalence
Source: PLoS One. 2013 Nov 26;8(11):e80299. doi: 10.1371/journal.pone.0080299 (PMC3841168; doi:10.1371/journal.pone.0080299)
Supplement: Table S3 — Mean bacterial DNA detected (Log genome equivalents/mL) in faecal samples by type of infant breastfeeding (n = 120). *T Student test p<0.05. (DOCX) [file pone.0080299.s003.docx]

**Table S3.** Mean bacterial DNA detected (Log genome equivalents/mL) in faecal samples by type of infant breastfeeding (n=120)

| **Bacterial group** | | **Exclusive Breastfeeding** | | **Complementary** | | **P*** |
| --- | --- | --- | --- | --- | --- | --- |
|  |  | **Mean** | **SD** | **Mean** | **SD** |  |
| *Lactobacillus* |  | 4.974 | 2.429 | 5.738 | 1.703 | 0.974 |
| *Bifidobacterium* |  | 9.813 | 1.338 | 10.003 | 1.011 | 0.396 |
| *Bacteroides* |  | 4.198 | 2.760 | 6.216 | 2.104 | **0.001** |
| *Staphylococcus* | *S. epidermidis* | 6.409 | 2.394 | 4.254 | 2.301 | **0.001** |
|  | *S. aureus* | 4.917 | 2.037 | 3.179 | 2.249 | **0.001** |
| *Streptococcus* |  | 6.275 | 0.911 | 5.962 | 0.885 | **0.042** |
| *Enterococcus* |  | 5.652 | 1.192 | 5.962 | 1.049 | 0.920 |
| *Clostridium leptum* |  | 3.395 | 2.118 | 3.927 | 2.384 | 0.254 |
| *Clostridium coccoides* |  | 4.302 | 1.800 | 4.964 | 1.983 | 0.091 |
| *Total Bacteria* |  | 9.811 | 0.687 | 9.775 | 0.782 | 0.407 |

*****T Student test p<0.05
