# Supplementary material for: Breast Milk and Gut Microbiota in African Mothers and Infants from an Area of High HIV Prevalence
Source: PLoS One. 2013 Nov 26;8(11):e80299. doi: 10.1371/journal.pone.0080299 (PMC3841168; doi:10.1371/journal.pone.0080299)
Supplement: Table S4 — Mean bacterial DNA detected (Log genome equivalents/mL) in faecal samples by infant nutritional status. *T Student test p<0.05. (DOCX) [file pone.0080299.s004.docx]

**Table S4.** Mean bacterial DNA detected (Log genome equivalents/mL) in faecal samples by infant nutritional status

| **Bacterial group** | | **Not Malnourished**  **(n=83)** | | **Malnourished**  **(n=37)** | | **P*** |
| --- | --- | --- | --- | --- | --- | --- |
|  |  | **Mean** | **SD** | **Mean** | **SD** |  |
| *Lactobacillus* |  | 5.482 | 1.873 | 5.590 | 2.178 | 0.782 |
| *Bifidobacterium* |  | 9.963 | 1.088 | 9.915 | 1.186 | 0.828 |
| *Bacteroides* |  | 5.577 | 2.335 | 5.74 | 2.810 | 0.742 |
| *Staphylococcus* | *S. epidermidis* | 4.726 | 2.570 | 5.234 | 2.394 | 0.310 |
|  | *S. aureus* | 3.923 | 2.258 | 3.156 | 2.498 | 0.099 |
| *Streptococcus* |  | 5.942 | 0.960 | 6.305 | 0.695 | **0.041** |
| *Enterococcus* |  | 5.782 | 1.119 | 6.074 | 1.032 | 0.179 |
| *Clostridium leptum* |  | 3.428 | 2.204 | 4.541 | 3.742 | **0.014** |
| *Clostridium coccoides* |  | 4.376 | 1.843 | 5.655 | 1.909 | **0.001** |
| *Total Bacteria* |  | 9.723 | 0.798 | 9.924 | 0.628 | 0.178 |

*****T Student test p<0.05
